# Electrocardiographic findings in pregnant women in Angola

**DOI:** 10.1111/anec.12980

**Published:** 2022-07-15

**Authors:** Mauer A. A. Gonçalves, João Mário Pedro, Carina Silva, Pedro Magalhães, Miguel Brito

**Affiliations:** ^1^ Centro de Estudos Avançados em Educação e Formação Médica (CEDUMED) Luanda Angola; ^2^ Faculty of Medicine University Agostinho Neto Luanda Angola; ^3^ Centro de Investigação em Saúde de Angola (CISA) Caxito Angola; ^4^ EPIUnit, Instituto de Saúde Pública Universidade do Porto Porto Portugal; ^5^ Health and Technology Research Center (H&TRC) Escola Superior de Tecnologia da Saúde de Lisboa, Instituto Politécnico de Lisboa Lisboa Portugal; ^6^ CEAUL ‐ Centro de Estatística e Aplicações, Faculdade de Ciências Universidade de Lisboa Lisboa Portugal

**Keywords:** Angola, electrocardiographic findings, normal pregnancy

## Abstract

**Background:**

Studies on the electrocardiogram findings in African pregnant women are limited. There is no information available in the literature on the electrocardiographic parameters of pregnant Angolan women.

**Objectives:**

The aim of this study was to describe electrocardiographic findings in women with normal pregnancies in Bengo Province, Angola.

**Methods:**

This is a community‐based study with a cross‐sectional design conducted between September 2013 and March 2014 in Bengo. The study involved 114 black pregnant women, compared with a paired control group comprising of 120 black non‐pregnant women, aged 15 to 42 years. A 12‐lead electrocardiogram and a rhythm strip were recorded for all participants.

**Results:**

In this study, the mean age was 26.2 ± 7.3 years. Comparing pregnant women vs. non‐pregnant, we found the following mean values: Heart rate (83 bpm vs. 74 bpm, *p* < .001), PR interval (146 ms vs. 151 ms, *p* = .034), QT interval (360 ms vs. 378 ms, *p* < .001), QTIc Fridericia (398 ms vs. 403, *p* = .017), QTIc Framingham (399 ms vs. 404 ms, *p* = .013) and T‐wave axis (34^0^ vs. 41^0^, *p* = .001).The main electrocardiographic changes found were: Sinus tachycardia (4.4% vs. 2.5%), T‐wave inversion (14.9% vs. 1.7%), Minor ST segment depression (4.5% vs. 0%) and left ventricular hypertrophy (11.4% vs. 11.7%, *p* = .726).

**Conclusions:**

Pregnant Angolan women compared with controls, had several significantly higher values for heart rate, and significantly lower values of systolic blood pressure and diastolic blood pressure, PR interval, QT interval, QTc interval by Fridericia and Framingham and T‐wave axis. Sinus tachycardia, T‐wave inversion, and left ventricular hypertrophy, were the main electrocardiographic changes found.

## Graphical abstract

Angolan women, compared with their non‐pregnant peer controls, displayed:

- higher values for heart rate,
- significantly lower levels of systolic blood pressure and diastolic blood pressure, PR intervals, QT intervals and QTc intervals according to Fridericia and Framingham and the T‐wave axis.

The main electrocardiographic changes found are:

- Sinus tachycardia, T‐wave inversion, minor ST segment depression and left ventricular hipertrophy.

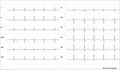

## 1 INTRODUCTION

Pregnancy is a physiological state characterized by adaptive changes that guarantee the adequate means for fetal development (Sanghavi & Rutherford, 2014). The cardiovascular (CDV) system undergoes progressive changes during pregnancy, resulting in hemodynamic changes characteristic of that period. The main changes involve increased blood volume, cardiac output, and decreased systemic vascular resistance and vascular reactivity (Metcalfe et al., 1981; Sanghavi & Rutherford, 2014).

The heart changes its size during pregnancy. The upward displacement of the diaphragm, caused by the swelling uterus, causes the heart to shift to the left and the anterior, so that the ictus cordis is palpated higher and laterally in the chest. These alterations, together with other physiological changes specific to pregnancy, can lead to different findings on the electrocardiogram (ECG), which must be taken into account to avoid errors of interpretation and exaggeration in subsequent assessment (Ayoub et al., 2002; Ciliberto et al., 2008).

The ECG, a simple, low‐cost test, has no deleterious effects on the fetus and should be requested whenever there are cardiovascular symptoms in pregnant women with suspected heart disease; especially with cardiac arrhythmias, the ECG is mandatory for the assessment of the prognosis or effectiveness of therapy (Ciliberto et al., 2008; Goloba et al., 2010).

Studies on the ECG findings in African pregnant women are limited, especially in sub‐Saharan Africa (SSA). Akinwusi et al. (2011), in a cross‐sectional study conducted in Nigeria, analyzed 69 normal pregnant and 70 healthy non‐pregnant controls, concluded that ECG changes showed a normal frontal‐plane QRS axis, normal PR interval, significantly rare normal Negroid pattern ST elevation, significant LVH based on Araoye RI >12 mm and a rarity of all forms of arrhythmias (Akinwusi et al., 2011). Salisu and Karaye (2010), in a cross‐sectional study conducted in a rural pregnant Nigerian women population, which included 123 pregnant subjects and 115 non‐pregnant controls, identified several differences in ECG resting, namely significantly lower QRS duration, and P, QRS, and T‐wave axes (Salisu & Karaye, 2010).

There is no information available in the literature on the parameters of electrocardiograms in Angolan women with normal pregnancies. Although there are some data in some African populations, local studies are needed, given the variation in genetics, environmental and behavioral conditions of the different population groups.

Using the data collected in the CardioBengo Study (Pedro et al., 2016), the aim of this study was to describe electrocardiographic findings in women with normal pregnancies, compared with a paired control group of non‐pregnant women in Bengo Province, Angola.

## 2 METHODS

The CardioBengo was a community‐based study with a cross‐sectional design conducted between September 2013 and March 2014 in the Municipality of Dande, Bengo, and selected a representative random sample of 2499 black individuals, stratified by sex and age between 15 and 84 years (Pedro et al., 2016).

In total, 234 women were included in the final analysis, aged 15 and 42 years of namely 114 apparently healthy pregnant women and 120 healthy non‐pregnant women within the same age group were selected from the total sample in order to constitute the non‐pregnant control group.

We collected information about age, education, alcohol and tobacco consumption through a structured interview conducted by trained and certified interviewers following the standardized protocol of the World Health Organization (WHO), based on the Surveillance Manual (STEPS) for Risk Factors for Chronic Diseases (central and expanded version 3.0) (WHO, 2015). We measured blood pressure, glucose and cholesterol levels in addition to the collection of medical history and anthropometric data.

The following risk factors for CVD were considered: participants who reported being on some antihypertensive medication or who presented mean values of systolic blood pressure (SBP) ≥140 mmHg and/or diastolic blood pressure (DBP) ≥90 mmHg were classified as hypertensive; participants with a defined diagnosis or treatment for Diabetes or fasting glycemia >126 mg/dl or postprandial glycemia >200 mg/dl) were considered diabetic; participants with total cholesterol levels >240 mg/dl (6.2 mmoL/L) or undergoing statins were considered with Hypercholesterolemia; participants with body mass index ≥30 kg/m^2^ were considered obese; with participants who currently smoked classified as smokers.

For the analyzes described in the present work, an exclusion criteria was previous or current heart failure, history suggestive of congenital or valvular heart disease, diabetes mellitus, hypertension, stroke, thyrotoxicosis, sickle cell disease and anemia.

### 2.1 Electrocardiographic recording

A 12‐lead electrocardiogram and a rhythm strip were recorded for all participants using a 12‐channel AsCARD Mr.Gray V 201 electrocardiograph (ASPEL, Zabierzów, Poland). The examination was performed in a private way behind closed curtains, with the individual at rest, in supine position, respecting the participant's rights to privacy. The exam was digitally recorded using cardio TEKA v001 database software (ASPEL, Zabierzów, Poland), and subsequently transferred to the Central Electrocardiography Laboratory at the University of Glasgow, where they were analyzed and processed by the University of Glasgow software and coded by the Minnesota Code (Prineas et al., 1982).

For the present study, P, T and QRS complex durations, PR and QT intervals, and P, T and QRS complex wave axes were automatically measured. The QT interval was corrected by the formula of Hodges, Bazett, Fridericia and Framingham. The Sokolow‐Lyon (S‐L) index (SV1 or SV2+RV5 or V6) was calculated, and left ventricular hypertrophy (LVH) was considered when the index ≥3.5 mV or 35 mm was determined (Sokolow & Lyon, 1949). The MC classified the electrocardiograms as presenting major, minor alterations or absence of abnormalities. The Normal limits of the ECG, as well as ECG abnormalities in this population were published elsewhere (Gonçalves et al., 2020, 2021, 2022). In view of the presence of alterations, abnormal ECGs were manually reviewed by two cardiologists, in order to guarantee the quality of the coding.

### 2.2 Statistical analysis

Means and standard deviations were computed and presented for quantitative variables and medians and interquartile range (IQR) was obtained for skewed data. Data were analyzed considering pregnant and non‐pregnant groups and it was also compared some parameters by the trimester pregnancy.

Student's *t*‐test, proportions test for two independent populations, and Chi square (*χ* ^2^) tests or, one‐way ANOVA and non‐parametric Kruskal‐Wallis tests were used for comparison between groups as appropriate. Bonferroni adjustments were used for multiple testing. The significance level was set at .05 and all statistical analysis was carried out using the software IBM SPSS® (Statistical Package for the Social Sciences) version 26.

## 3 RESULTS

Table 1 shows the baseline characteristics of the 234 individuals (114 pregnant and 120 non‐pregnant). The most representative age group was 20–35 years (65.4%) and the least represented age group was ≥35 years ((12.8%). Considering all participants, the mean age was 26.2 ± 7.3 years. As expected, the pregnant women had significantly higher values for body weight, BMI, waist circumference, hip circumference and waist‐to‐hip ratio, while non‐pregnant women had higher systolic blood pressure (SBP) and diastolic blood pressure values (DBP).

**TABLE 1:** Physical, hemodynamic, and biochemical parameters in pregnant and non‐pregnant (mean [SD] for continuous parameters)

Parameters	Pregnant *n* = 114	Non‐pregnant *n* = 120	*p*	Total *N* = 234
Age group (years)				
<20	20.2%	23.3%	.999^a^	21.8%
20–35	67.5%	63.3%	.999^a^	65.4%
>35	12.3%	13.3%	.999^a^	12.8%
Age (years)	26.4 (7.1)	26 (7.4)	.692^b^	26.2 (7.3)
Body weight (kg)	60.5 (11.0)	55.4 (11.8)	**.001** ^b^	57.9 (11.7)
Height (cm)	1.6 (0.06)	1.6 (0.07)	.997^b^	1.6 (0.07)
BMI (Kg/m^2^)	24.9 (4.1)	22.8 (4.7)	**<.001** ^b^	23.8 (4.5)
Waist circumference (cm)	88.5 (11.6)	75.9 (11.5)	**<.001** ^b^	82 (13.1)
Hip circumference (cm)	96.6 (8.6)	92.3 (9.7)	**<.001** ^b^	94.4 (9.4)
Waist‐hip‐ratio	0.91 (.08)	0.82 (.06)	**<.001** ^b^	0.87 (.09)
SBP (mmHg)	101 (12)	110 (14.6)	**<.001** ^b^	105.6 (14.1)
DBP (mmHg)	63.7 (8.1)	72.2 (11.7)	**<.001** ^b^	68.1 (10.9)
Glycemia (mg/dl)	97.4 (13.7)	101.2 (17)	.064^b^	99.3 (15.6)
Total cholesterol (mg/dl)	201.6 (33.8)	174.8 (28.6)	**<.001** ^b^	189.2 (34.1)

^a^ Test for the difference between two independent population proportions, *p*‐values adjusted by the Bonferroni method. Bold values correspond to *p* < .001.

^b^ *T*‐test for independent populations.

The ECG parameters for pregnant and non‐pregnant participants are presented in Table 2. The non‐pregnant had significantly higher mean values for PR interval (146 ms vs. 151 ms, *p* = .034), QT interval (360 ms vs. 378 ms, *p* < .001), QTIc Fridericia (398 ms vs. 403, *p* = .017), QTIc Framinghan (399 ms vs. 404 ms, *p* = .013) and T‐wave axis (34^0^ vs. 41^0^, *p* = .001), while pregnant women had a significantly high value only for heart rate (HR) (83 bpm vs. 74 bpm, *p* < .001). Differences in other comparisons were not statistically significant.

**TABLE 2:** ECG parameters in pregnant and non‐pregnant (mean [SD])

ECG parameters	Pregnant *n* = 114	Non‐pregnant *n* = 120	*p* *
HR (bpm)	83 (12)	74 (11.6)	**<.001**
P‐wave duration (ms)	105 (9.3)	107 (10.5)	.288
PRI duration (ms)	146 (20.8)	152 (20.1)	**.034**
QRS duration (ms)	84 (6.2)	86 (7.3)	.053
QTI duration (ms)	360 (24.1)	379 (24.9)	**<.001**
QTIc, Hodges (ms)	399 (16.1)	403 (14.7)	.093
QTIc, Bazett (ms)	419 (20.9)	417 (18.7)	.352
QTIc, Fridericia, (ms)	398 (17.6)	403 (15.4)	**.017**
QTIc, Framinghan, (ms)	399 (16.3)	404 (14.7)	**.013**
P‐wave axis (°)	48 (22.8)	46 (27.4)	.444
QRS axis (°)	44 (19.1)	48 (20.8)	.120
T‐wave axis (°)	34 (15.9)	41 (15.8)	**.001**

* *T*‐test for independent populations. Bold values correspond to *p* < .001.

Table 3 compares ECG parameters among pregnant subjects grouped by pregnancy trimester (1st, 2nd and 3rd). It shows that mean P‐wave duration was significantly reduced as pregnancy advanced from 1st to 3rd trimester (110 ms; 106 ms; 102 ms; *p* = .010), while differences in other comparisons were not statistically significant.

**TABLE 3:** Distribution of ECG parameters (mean (SD) or median (IQR) if normality is not verified in pregnant group according to trimester of pregnancy

Parameters	1st trimester *n* = 28	2nd trimester *n* = 43	3rd trimester *n* = 43	*p*
HR (bpm)	80.1 (12.9)	83.42 (12.3)	83.16 (11.16)	.488^a^
P‐wave duration (ms)	110 (8)	106 (10)	102 (14)	**.010** ^b^
PRI duration (ms)	146 (25)	146 (30)	136 (26)	.053^b^
QRS duration (ms)	86 (6.6)	84 (6.4)	83 (5.5)	.097^a^
QTI duration (ms)	366 (26.0)	362 (25.9)	355 (19.9)	.133^a^
QTIc, Hodges (ms)	400 (16.7)	403 (13.9)	395 (17.0)	.076^a^
QTIc, Bazett (ms)	419 (22.0)	423 (17.5)	415 (22.9)	.199^a^
QTIc, Fridericia, (ms)	400 (18.3)	401 (16.2)	394 (17.9)	.104^a^
QTIc, Framinghan, (ms)	401 (17.1)	402 (14.9)	395 (16.4)	.092^a^
P‐wave axis (°)	59 (19)	52 (41)	55 (25)	.418^b^
QRS axis (°)	45 (24)	51 (25)	43 (24)	.537^b^
T‐wave axis (°)	33 (21)	38 (19)	37 (19)	.202^b^

^a^ One‐way ANOVA. Bold values correspond to *p* < .001.

^b^ Non‐parametric Kruskal‐Wallis test.

Table 4 gives information about ECG changes in pregnant and non‐pregnant participants. All these changes were found to be minor according to the Minnesota code: The main changes found were: sinus bradycardia (0% vs. 6.6%), sinus tachycardia (4.4% vs. 2.5%), first degree AV block (1.8% vs. 0%), T‐wave inversion (14.9% vs. 1.7%), minor ST segment depression (4.5% vs. 0%), incomplete right bundle branch block (0.9% vs. 0%), atrial premature contractions (APCS) and ventricular premature contractions (VPCS) together (0.9% vs. 0%), and LVH (11.4% vs. 11.7%, *p* = .726).

**TABLE 4:** ECG changes in pregnant and non‐pregnant

	Pregnant	Non‐pregnant	*p*
Sinus bradycardia	0	8 (6.6%)	**–**
Sinus tachycardia	5 (4.4%)	3 (2.5%)	**–**
First degree AV block	2 (1.8)	0	**–**
T‐wave inversion: anteroseptal site (V2–V4)	14 (12.3%)	2 (1.7%)	**–**
T‐wave inversion: inferior site (DII, DII e aVF)	3 (2.6%)	0	**–**
Minor ST segment depression: site anteroseptal (V2–V4)	2 (1.8%)	0	**–**
Minor ST segment depression: site inferior (DII, DIII e aVF)	2 (1.8%)	0	**–**
Minor ST segment depression: site lateral (DI, aVL, V5 e V6)	1 (0.9%)	0	**–**
Incomplete right bundle branch block	1 (0.9%)	0	**–**
APCS and VPCS together	1 (0.9%)	0	**–**
LVH	13 (11.4%)	14 (11.7%)	**.726**

Abbreviations: APCS, atrial premature contractions; LVH, left ventricular hypertrophy; VPCS, ventricular premature contractions. Bold values correspond to *p* < .001.

## 4 DISCUSSION

In the present study we analyze the electrocardiographic findings in Angolans with normal pregnancies in comparison with a paired control group of non‐pregnant women. These data came from the population of the municipality of Dande, province of Bengo, in northern Angola. The electrocardiographic changes found can be explained by the physiological adaptations that occur during normal pregnancy.

Our study has shown that pregnant women had significantly higher values for heart rate, and significantly lower SBP, DBP, PR interval, QT interval, and QTc interval by Fridericia and Framingham and T‐wave axis.

It was found that pregnant women had significantly lower values of systolic and diastolic blood pressure. These results agree with the results of several studies (Mahendru et al., 2014; Salisu & Karaye, 2010). This may be explained by the hormonal alterations that occur throughout pregnancy, as pregnancy has an impact on the reduction of systemic vascular resistance and afterload, due to peripheral vasodilation and low resistance, with high blood flow to the uterus and placenta, leading to many hemodynamic changes including a fall in blood pressure (Clapp & Capeless, 1997; Maroo & Raymond, 2007; Sanghavi & Rutherford, 2014). These interactions involve the renin‐angiotensin‐aldosterone system, the reproductive hormones, prostaglandins, nitric oxide and atrial natriuretic peptide (McAnulty et al., 2004; Sanghavi & Rutherford, 2014).

In the present study, it was found that all participants were in sinus rhythm. The pregnant women had a significantly high value for HR, which is in line with several existing studies (Akinwusi et al., 2011; Grindheim et al., 2012; Mahendru et al., 2014; Salisu & Karaye, 2010). In pregnant women, due to physiological reasons, the heart rate increases by an average of about 15 beats/min (or approximately 20% above the baseline HR), reaching its maximum from the 32nd week until the end of pregnancy (Duvekot & Peeters, 1994; Maroo & Raymond, 2007; Meah et al., 2016).

The PR interval in the present study was significantly higher in non‐pregnant participants than in pregnant ones—a Nigerian study revealed a slightly elevated PR interval in non‐pregnant women, but without a statistically significant difference, while an Indian study showed results similar to ours (Nandini et al., 2011; Salisu & Karaye, 2010). The PR interval increases with the reduction of HR and varies with age and body mass index. This increase is due to cardiac conduction abnormalities caused by the stretching of the chambers, mainly in the left atrium, as well as the influence of parasympathetic/sympathetic tone, which explain this finding (Carruth et al., 1981; Nandini et al., 2011; Nikolaidou et al., 2016; Soliman & Rautaharju, 2012).

The QRS duration represents the ventricular depolarization. In our study, the QRS duration was slightly higher in controls than in pregnant women, while in other studies it was also found that the QRS was significantly higher in controls (Carruth et al., 1981; Edemeka & Ekong, 1995; Salisu & Karaye, 2010).

The QT interval represents the time taken for depolarization and repolarization in the ventricular myocardium. It should be noted that the QTc interval represents complex and interrelated aspects of cardiac electrophysiology, cardiac geometry, torso shape, tissue impedance, and biological signal processing (Ciliberto et al., 2008; Hunter & Robson, 1992; Nandini et al., 2011, 2014). In the present study, The QT interval was higher in non‐pregnant women compared with the pregnant subjects, results that are different from other studies (Carruth et al., 1981; Nandini et al., 2014).

The QT interval was corrected for heart rate by the formula of Hodges, Bazett, Fridericia, and Framingham, but only the QTc interval by Fridericia and Framingham showed statistically significant high values in controls. Although most studies do not reveal the method used to correct the QT interval, these results are similar to the study by Salisu and Karaye (2010) and different from other studies, in which the QTc is higher in pregnant subjects (Carruth et al., 1981; Lechmanová et al., 2002; Nandini et al., 2014; Ozmen et al., 2006; Salisu & Karaye, 2010).

QT interval prolongation has been associated with an increased risk of cardiac arrhythmias, including Torsades de Pointes (Luo et al., 2022; Zamani et al., 2014). Regarding the clinical implications of this finding, unlike other studies that showed prolongation of the QT interval in pregnancy, our study showed a reduction in this parameter, which suggests a reduced risk of arrhythmias in pregnant Angolan women. But studies with larger samples should be carried out.

Regarding the QRS axis, there was no significant difference between pregnant women and controls, findings that are not in accordance with several studies that show that there is a significant difference between pregnant and non‐pregnant women, suggesting that the QRS axis in pregnancy is more superiorly directed than the QRS axis in the controls (Carruth et al., 1981; Edemeka & Ekong, 1995; Goloba et al., 2010; Maroo & Raymond, 2007; Salisu & Karaye, 2010; Schwartz & Schamroth, 1979). The leftward axis deviation can be attributed to the horizontal heart position that occurs from the elevation and rotation of heart as a result of the enlarging uterus. Changes in left ventricular size and mass with associated increased volume may cause the apical impulse to be displaced to the left. Increased left ventricular load and blood volume also contribute to the shifting of the heart (Chia et al., 2002; Misra et al., 1986; Singh et al., 1986).

It was also observed that in non‐pregnant women there was a significant increase in the T‐wave axis. This was in line with previous studies (Carruth et al., 1981; Salisu & Karaye, 2010). In the majority of pregnant women there was a leftward deviation of the mean axis during pregnancy, particularly in the third trimester, but the range of variability of the axis was wide. The range of T‐vector changes is consistent with previous results of considerable variation in pregnant women, more than in non‐pregnant women (Carruth et al., 1981).

Regarding the groups by trimester, we observed that the duration of the P‐wave was significantly reduced as the pregnancy progressed from the 1st to the 3rd trimester. A study carried out in India showed no significant difference in P‐wave duration between pregnant women and the control group (Nandini et al., 2011).

In our study, the pregnant women had identical PR interval values in the 1st and 2nd trimester and a slight reduction in the 3rd trimester, other authors have also made similar conclusions (Madras & Challa, 2015; Nandini et al., 2011). The increased HR that accompanies pregnancy may be reflected in decrease of PR interval due to shortened atrioventricular conduction (Adamson & Nelson‐Piercy, 2008; Nandini et al., 2011).

There was a slight decrease in QRS duration in the 1st trimester compared to the 2nd and 3rd trimester, with no statistical difference; these results are in agreement with studies in Nigeria that did not observe any significant difference in the QRS durations between the trimesters (Edemeka & Ekong, 1995) and not in agreement with a study that revealed that QRS duration increased slightly in the late pregnancy (Carruth et al., 1981).

Regarding ECG changes, it was found that there were no pregnant women with sinus bradycardia in this study, while there were 6.6% of non‐pregnant women with sinus bradycardia. This study agrees with a Nigerian study that revealed that there were no pregnant women with sinus bradycardia and 5.7% of non‐pregnant women had sinus bradycardia (Akinwusi et al., 2011). A heart rate of less than 60 bpm is unusual during pregnancy and is usually found in healthy young women; it can also occur during sleep or in athletes in excellent aerobic condition (Ghorayeb et al., 2013; Metcalfe et al., 1981).

On the other hand, 4.4% of pregnant women and 2.5% of non‐pregnant women had sinus tachycardia. A study in Nigerians with a normal pregnancy showed that 8.7% of pregnant women and 2.9% of non‐pregnant women had sinus tachycardia (Akinwusi et al., 2011). HR increases during normal pregnancy, but not to the level of tachycardia. HR increases progressively throughout the pregnancy by 10 to 20 bpm, reaching the highest rate in the third trimester, a 20% to 25% increase over baseline (Grindheim et al., 2012; Mahendru et al., 2014).

We observed T‐wave inversion in 1.7% of non‐pregnant women and 14.9% of the pregnant women, with the most common leads involved being V2‐V4 (12.3%), DII, DIII, and aVF (2.6%). Minor ST segment depression were observed in 4.5% of pregnant women. An observational study revealed that inverted T‐waves were seen in 8.2% of cases during the second and third trimesters, and the inversion was predominantly observed in lead III and chest leads V1–V3 (Ananthakrishnan et al., 2020). The results of this study are in line with several studies showing significant T‐wave inversion in pregnant women (Ananthakrishnan et al., 2020; Carruth et al., 1981; Oram & Holt, 1961; Sunitha et al., 2014). It is possible that the T‐wave inversion is a temporary ischemia caused by the increased workload on the heart due to increased blood volume throughout pregnancy (Misra et al., 1986).

There were no significant differences in LVH using Sokolow‐Lyon criteria between pregnant and non‐pregnant women. These results differ from a study that showed significant higher LVH values in pregnant women based on Araoye criterion (Akinwusi et al., 2011). Previous studies in pregnant women have shown that the heart is enlarged by chamber dilatation and hypertrophy as a result of the hemodynamic changes that occur during this period, which can be seen during the second trimester and is more pronounced in late pregnancy (Ciliberto et al., 2008; Hunter & Robson, 1992).

It is important to highlight a limitation of this study, the cross‐sectional study design, meaning a causal relationship cannot be established. The small sample size is another limitation, as is the fact that the ECGs were obtained only once at the beginning of the study; ECG criteria can be dynamic and could be more significant if several ECGs were obtained at different time points. However, this study has several strengths: it is one of the first population‐based studies conducted in Angola, with a representative sample randomly selected; it is the largest study to date that identifies ECG findings in an autochthonous Angolan population; ECG collection, recording, and measurement were standardized, and the exams were performed by a single trained technician, and the ECG abnormalities were manually reviewed by two experienced cardiologists to guarantee the quality of the coding.

## 5 CONCLUSION

Our study suggests that the pregnant women had a significantly higher value for heart rate, and significantly lower values of systolic blood pressure and diastolic blood pressure, PR interval, QT interval, QTc interval by Fridericia and Framingham and T‐wave axis. Among pregnant subjects grouped by trimester, the study showed that mean P‐wave duration was significantly reduced as pregnancy advanced from 1st to 3rd trimester. Sinus tachycardia, T‐wave inversion, and left ventricular hypertrophy, were the main changes found in pregnant women. These results should be considered when interpreting the ECG in pregnant Angolan women. The understanding and recognition of possible electrocardiographic changes are essential in monitoring women throughout pregnancy.

## AUTHOR CONTRIBUTIONS

Mauer A. A. Gonçalves, João Mário Pedro, Miguel Brito, and Pedro Magalhães involved in conceptualization; João Mário Pedro and Miguel Brito involved in field work and data curation; Carina Silva and Pedro Magalhães involved in formal analysis and statistics; Mauer A. A. Gonçalves involved in first draft of the manuscript; Mauer A. A. Gonçalves, Carina Silva, Miguel Brito, and Pedro Magalhães involved in manuscript review and editing. All authors approved the manuscript final version.

## CONFLICT OF INTEREST

The authors declare that there is no conflict of interest regarding the publication of this article.

## ETHICS APPROVAL AND CONSENT TO PARTICIPATE

The Ethics Committee of the Angolan Ministry of Health approved the CardioBengo study protocol and all use of secondary data, and the present study was approved by the independent ethics committee of the Faculty of Medicine of Agostinho Neto University (DELIBERATION n° 9/2020). Written informed consent was obtained from all participants prior to data collection, following all human research standards according to the Helsinki declaration on the ethical principles for medical research involving human subjects.

## Data Availability

The data that support the findings of this study are available from the corresponding author upon reasonable request.
